# Supplementary material for: The histone chaperone Spn1 preserves chromatin protections at promoters and nucleosome positioning in open reading frames
Source: G3 (Bethesda). 2025 Feb 17;15(4):jkaf032. doi: 10.1093/g3journal/jkaf032 (PMC12005155; doi:10.1093/g3journal/jkaf032)
Supplement: jkaf032_Supplementary_Data [file jkaf032_supplementary_data.zip › File_S1_G3-2025-405650.pdf]

SUPPLEMENTARY DATA

Table S1: Plasmids and yeast strains used in this study.

**Plasmid**	**Description**	
pCR311	Full length wild type *SPN1* with 403 bp of upstream sequence and 116bp of downstream sequence, myc2 tagged at the amino terminus, pRS313 (CEN, *HIS3*)	
pCR312	*spn1*^{K192N} with 403 bp of upstream sequence and 116bp of downstream sequence, myc2 tagged at the amino terminus, pRS313 (CEN, *HIS3*)	
pAA344	*spn1*$^{141\text{-}305}$ with 403 bp of upstream sequence and 116bp of downstream sequence, myc2 tagged at the amino terminus, pRS313 (CEN, *HIS3*)	
**Strain**	**Genotype**	**Source**
BY4741	*MATa his3Δ1 leu2Δ0 met15Δ0 ura3Δ0*	Research Genetics
LZ0-1	BY4741 *spn1Δ::LEU2* + pCR311	(Li et al. 2018), (Zhang et al. 2008)
LZ0-2	BY4741 *spn1Δ::LEU2* + pCR312	(Li et al. 2018), (Zhang et al. 2008)
LZ0-3	BY4741 *spn1Δ::LEU2* + pAA344	(Li et al. 2018), (Zhang et al. 2008)

Figure S1

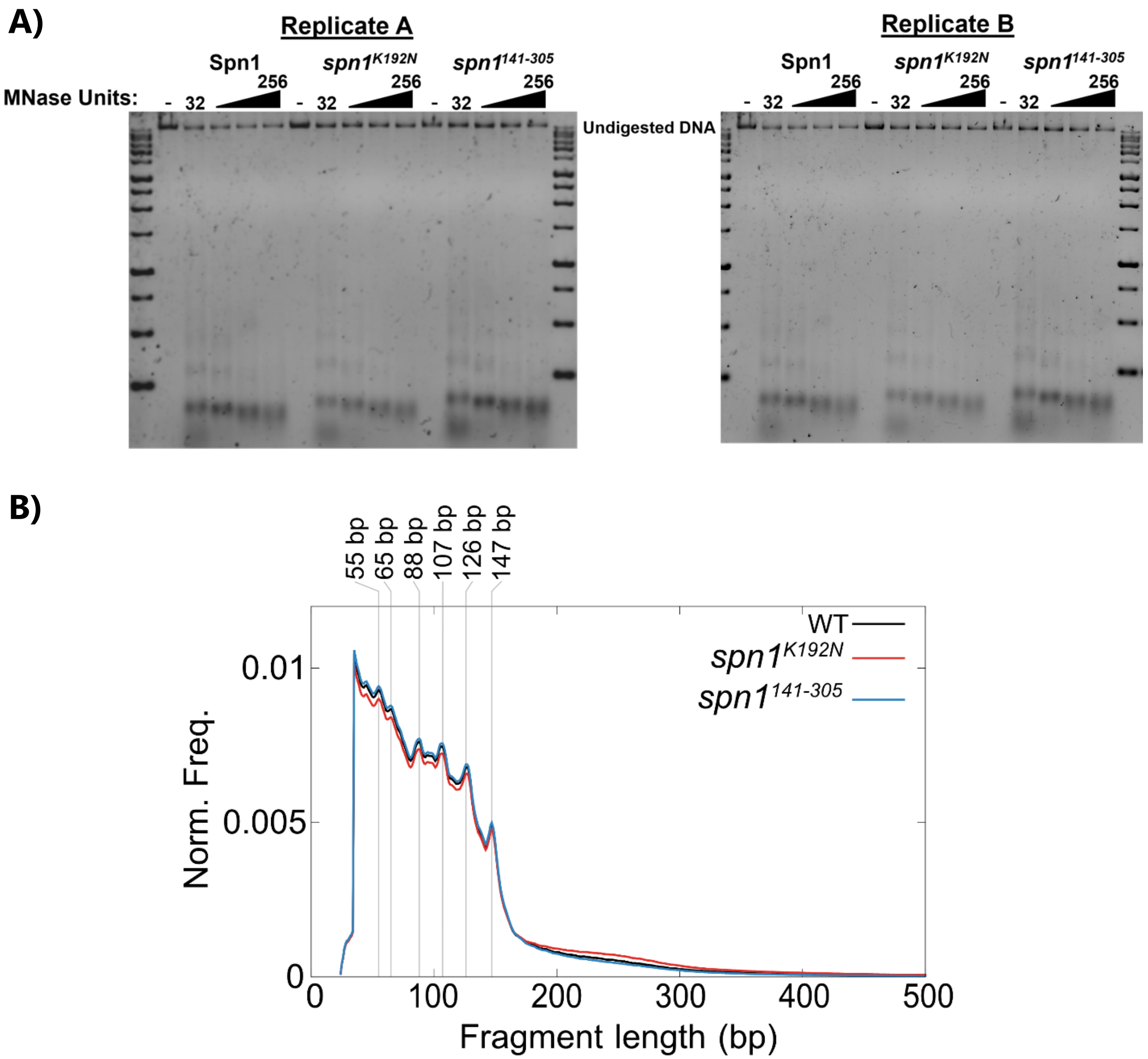

Figure S1: Characterization of MNase digested samples demonstrates uniform distribution of protected fragments. (A) Yeast nuclei isolated from yeast expressing wild-type Spn1, *spn1*^{K192N} and *spn1*$^{141\text{-}305}$ were digested with increasing amounts of MNase then subjected to agarose gel electrophoresis. (B) The normalized fragment length distribution of sequencing datasets generated from MNase digested samples (wild-type: black, *spn1*^{K192N}: red, *spn1*$^{141\text{-}305}$: blue). Fragment length distributions were normalized by averaging distributions from each sample followed by resampling.

Figure S2

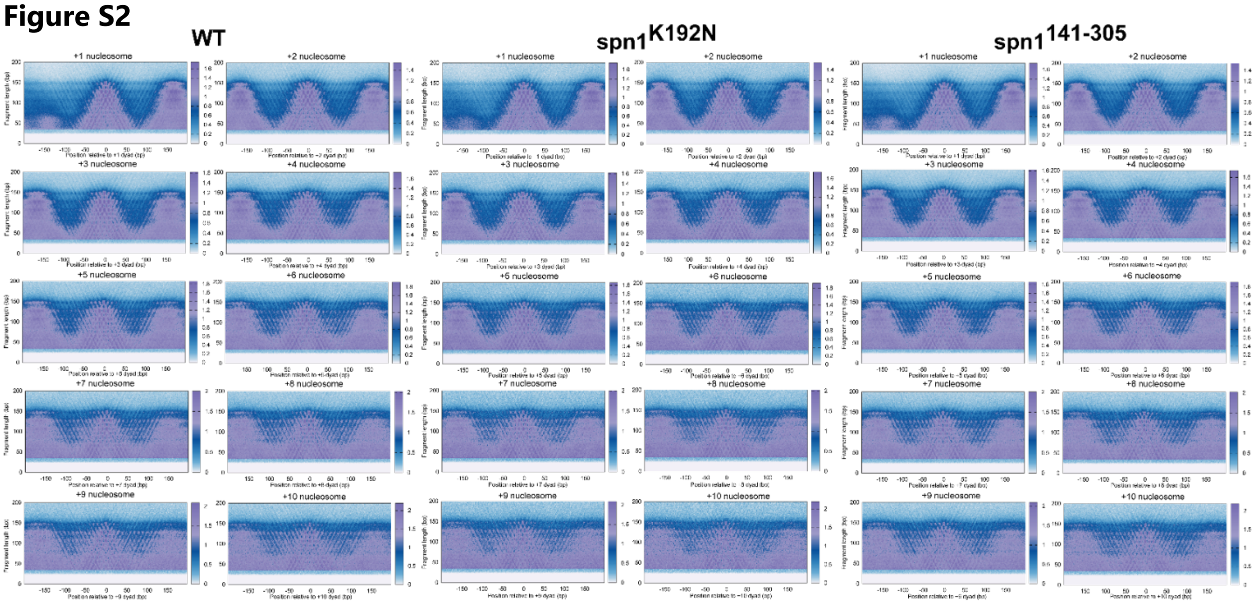

Figure S2: V-plots were produced by mapping fragment midpoints at each nucleosome dyad position from positions +1 to +10 over 4688 genes in wild-type or Spn1 mutant expressing yeast.

Figure S3

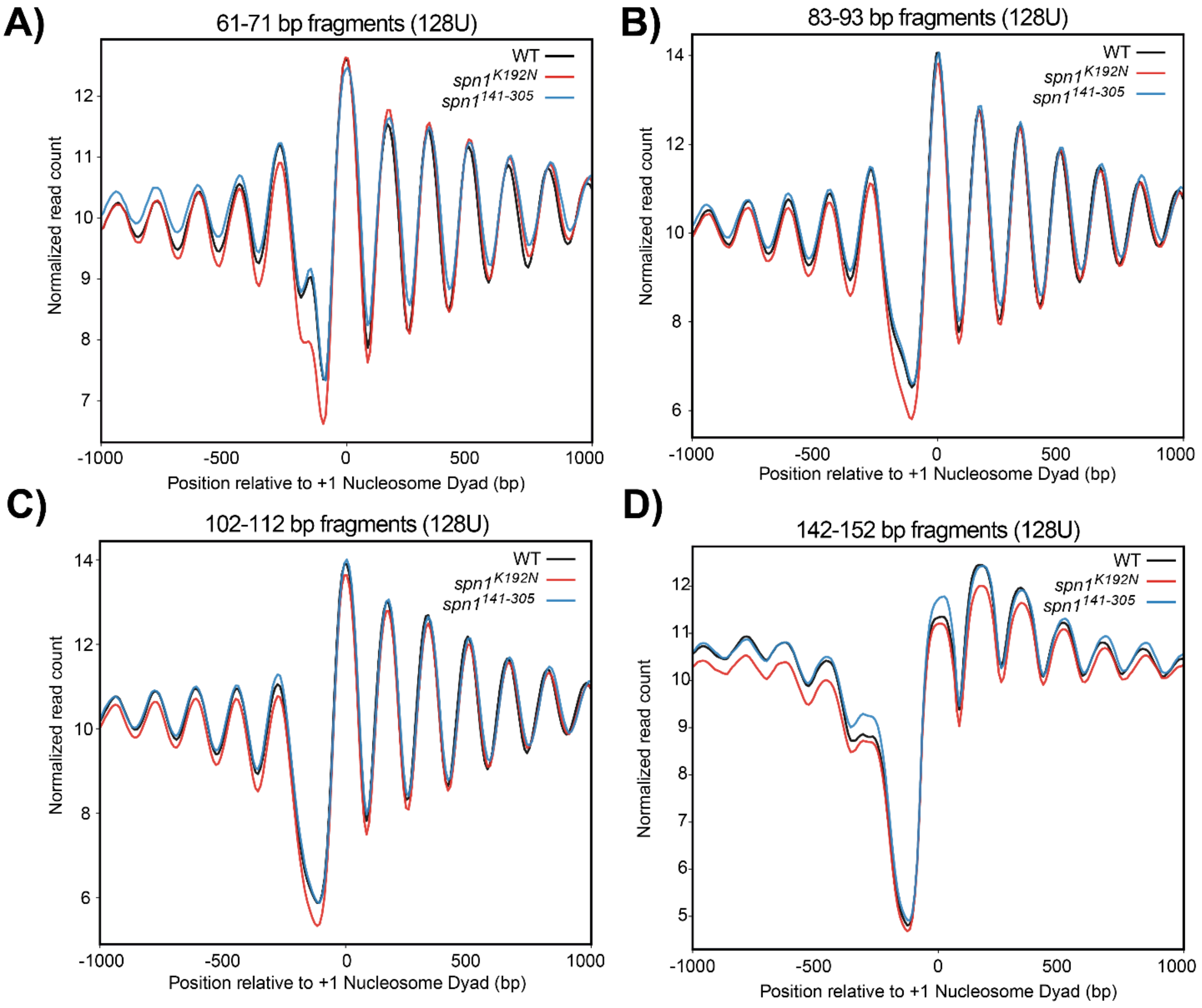

Figure S3: *spn1*^{K192N} cells exhibit a loss of MNase-protected fragments in NDRs, which diminishes as fragment length increases. Profiles of MNase protected fragments (A) 61-71 bp, (B) 83-93 bp, (C) 102-112 bp, and (D) 142-152 bp in length mapped over 4688 genes aligned to the +1 nucleosome dyad in wild-type, *spn1*^{K192N}, and *spn1*$^{141\text{-}305}$ expressing yeast.

Figure S4

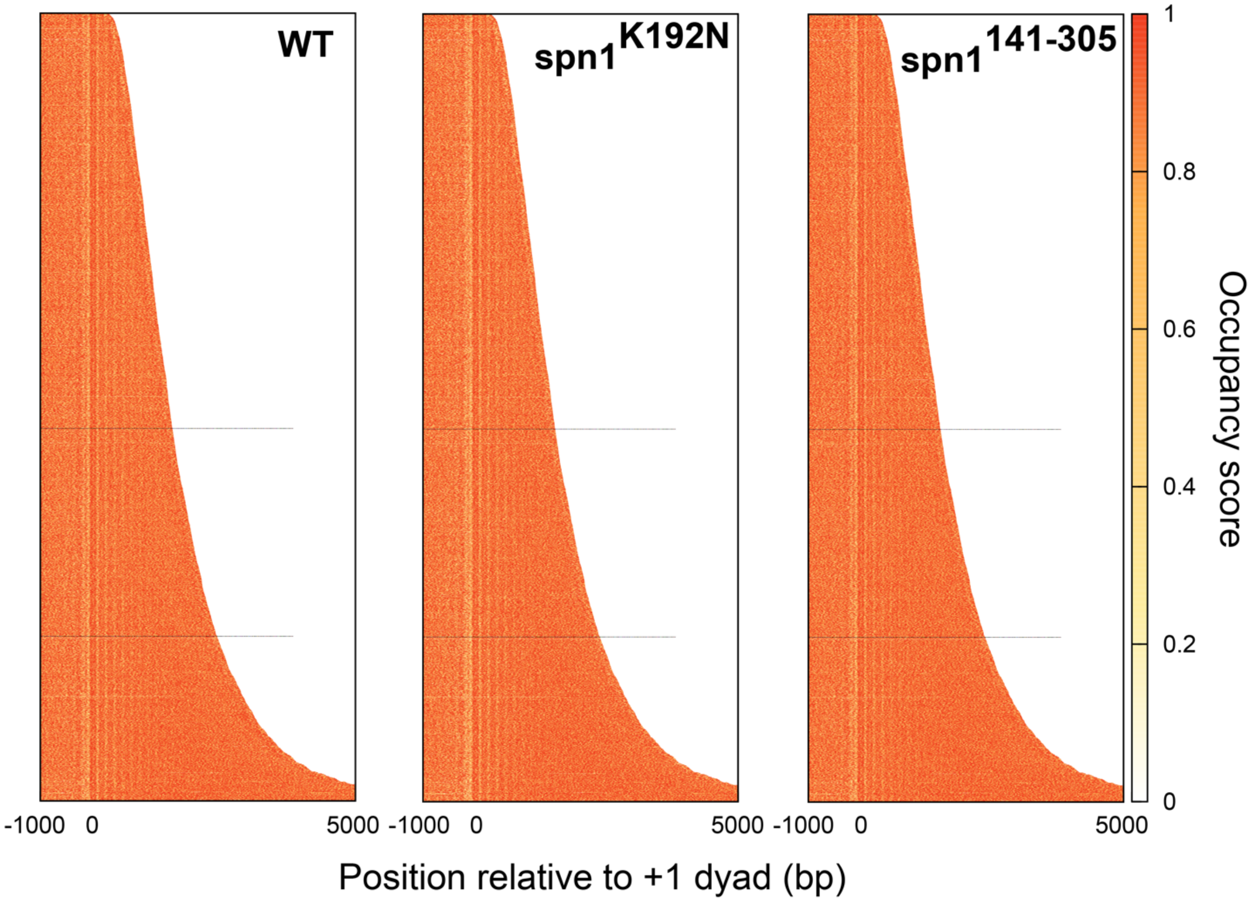

Figure S4: *spn1*^{K192N} cells experience a loss in short chromatin protections over NDRs genome wide. Heatmaps displaying occupancy of MNase protected fragments (0-60bp) across 4688 genes in wild-type, *spn1*^{K192N}, and *spn1*$^{141\text{-}305}$ expressing yeast.

Figure S5

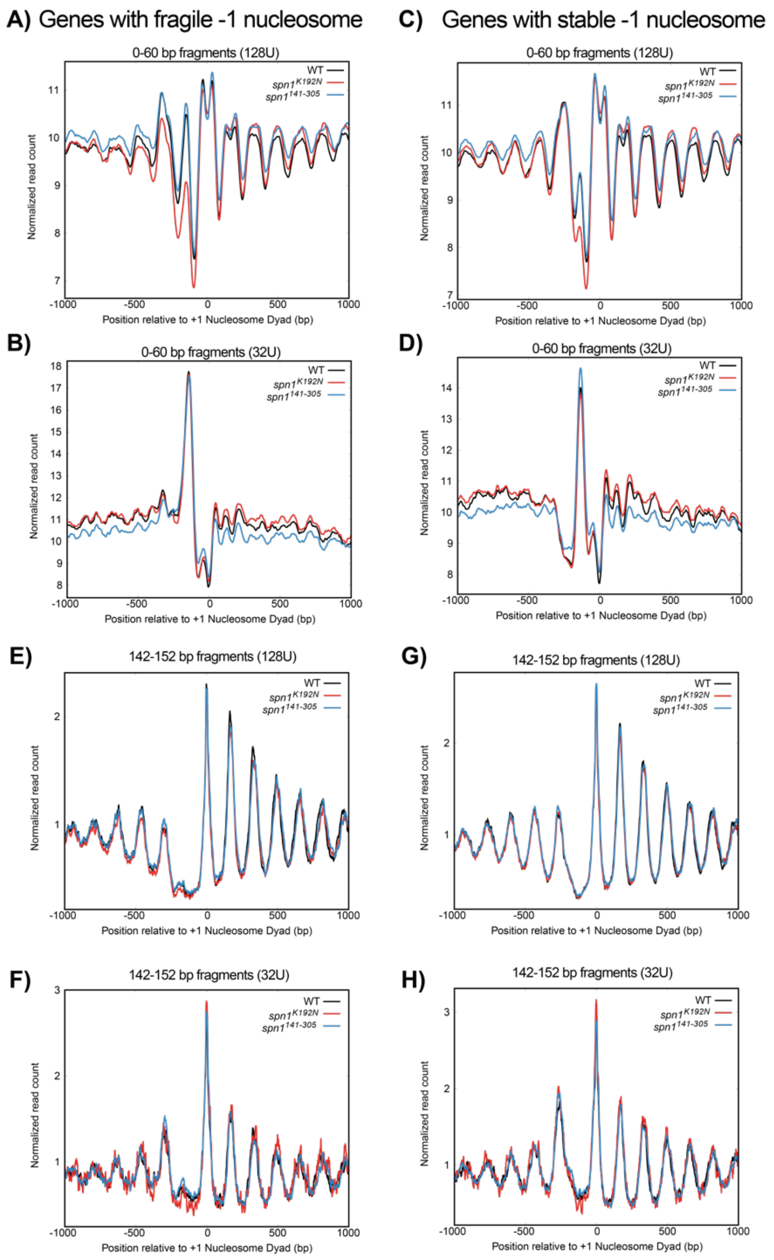

Figure S5: *spn1*^{K192N} cells exhibit a loss of MNase-protected fragments in NDRs with or without fragile nucleosomes. Profiles of MNase protected fragments 0-60 bp in length from (A) high MNase (128U) and (B) low MNase (32U) mapped over genes containing a fragile –1 nucleosome in NDRs as defined in Kubik et al. (Kubik et al. 2015) aligned to the +1 nucleosome dyad in wild-type, *spn1*^{K192N}, and *spn1*$^{141-305}$ expressing yeast. Profiles of MNase protected fragments 0-60 bp in length from (C) high MNase (128U) and (D) low MNase (32U) mapped

over genes containing a stable –1 nucleosome in NDRs as defined in Kubik *et al.* (Kubik et al. 2015) aligned to the +1 nucleosome dyad in wild-type, *spn1*^{K192N}, and *spn1*$^{141-305}$ expressing yeast. Profiles of MNase protected fragments 142-152 bp in length from (E) high MNase (128U) and (F) low MNase (32U) mapped over genes containing a fragile –1 nucleosome in NDRs as defined in Kubik et al. (PMID: 26545077) aligned to the +1 nucleosome dyad in wild-type, *spn1*^{K192N}, and *spn1*$^{141-305}$ expressing yeast. Profiles of MNase protected fragments 142-152 bp in length from (G) high MNase (128U) and (H) low MNase (32U) mapped over genes containing a stable –1 nucleosome in NDRs as defined in Kubik *et al.* (Kubik et al. 2015) aligned to the +1 nucleosome dyad in wild-type, *spn1*^{K192N}, and *spn1*$^{141-305}$ expressing yeast.

Figure S6

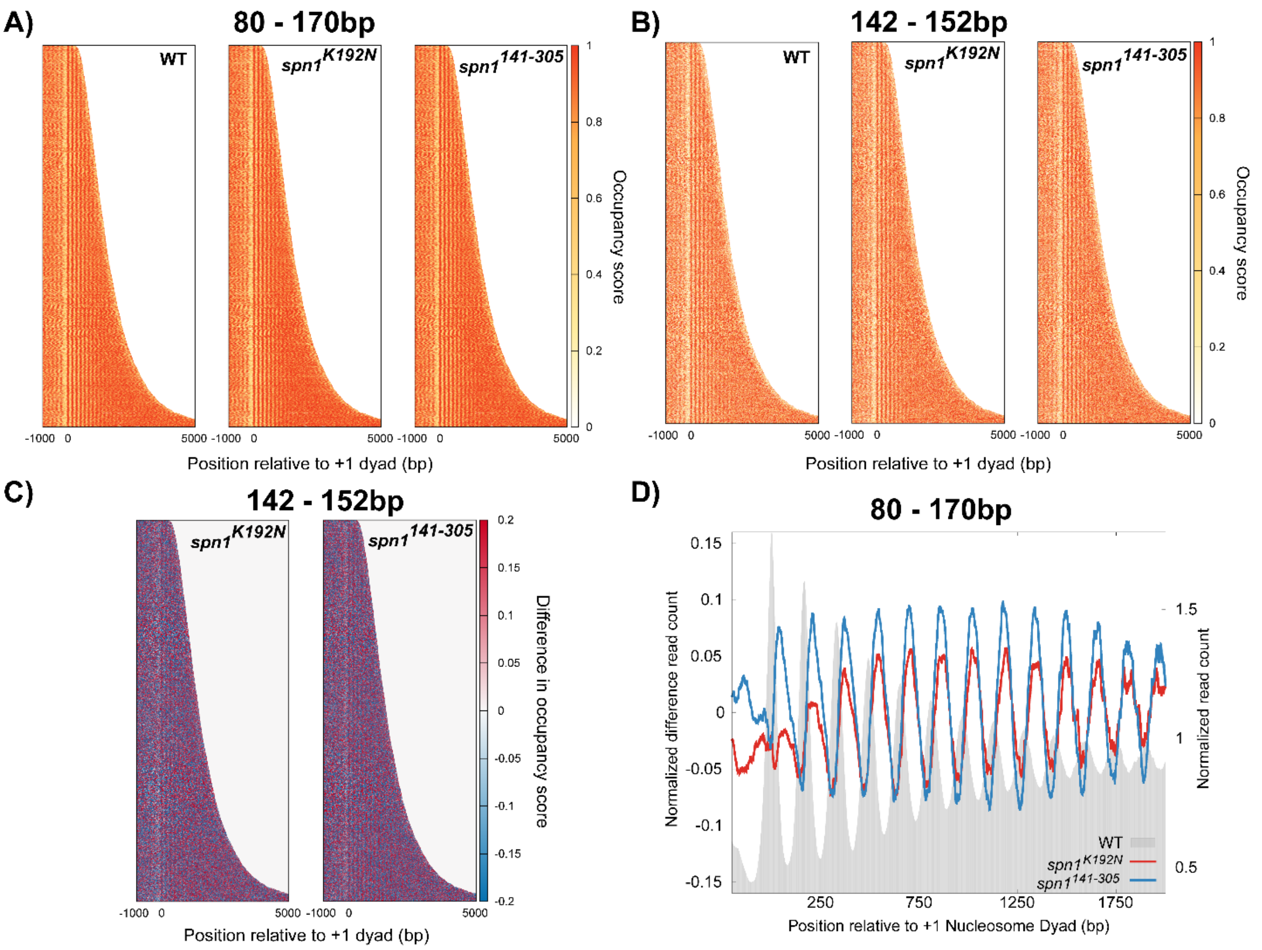

Figure S6: Mutations in Spn1 propagate a downstream shift in chromatin protections over open reading frames genome wide. (A) Heatmaps of occupancy of MNase protected fragments (80-170bp) across 4688 genes in wild-type, *spn1*^{K192N}, and *spn1*$^{141\text{-}305}$ expressing yeast. (B) Heatmap of occupancy of MNase protected fragments (142-152bp) across 4807 genes in wild-type, spn1^{K192N}, and spn1$^{141\text{-}305}$ expressing yeast. (C) Difference heatmap between wild-type and mutant Spn1 expressing yeast strains scored by changes in 142-152 bp fragment occupancy. Genes are aligned by their +1 nucleosome dyad position and ranked by length. Scores are calculated by subtracting wild-type occupancy data from mutant occupancy. Regions in red are enriched for fragments at that position while blue regions are depleted for fragments. (D) Difference profile plot between wild-type and mutant samples. Average nucleosome density from wild-type expressing yeast is plotted in grey. Differences between wild-type and mutants (*spn1*^{K192N}, red and *spn1*$^{141\text{-}305}$, blue) against wild-type replicates are plotted.

Figure S7

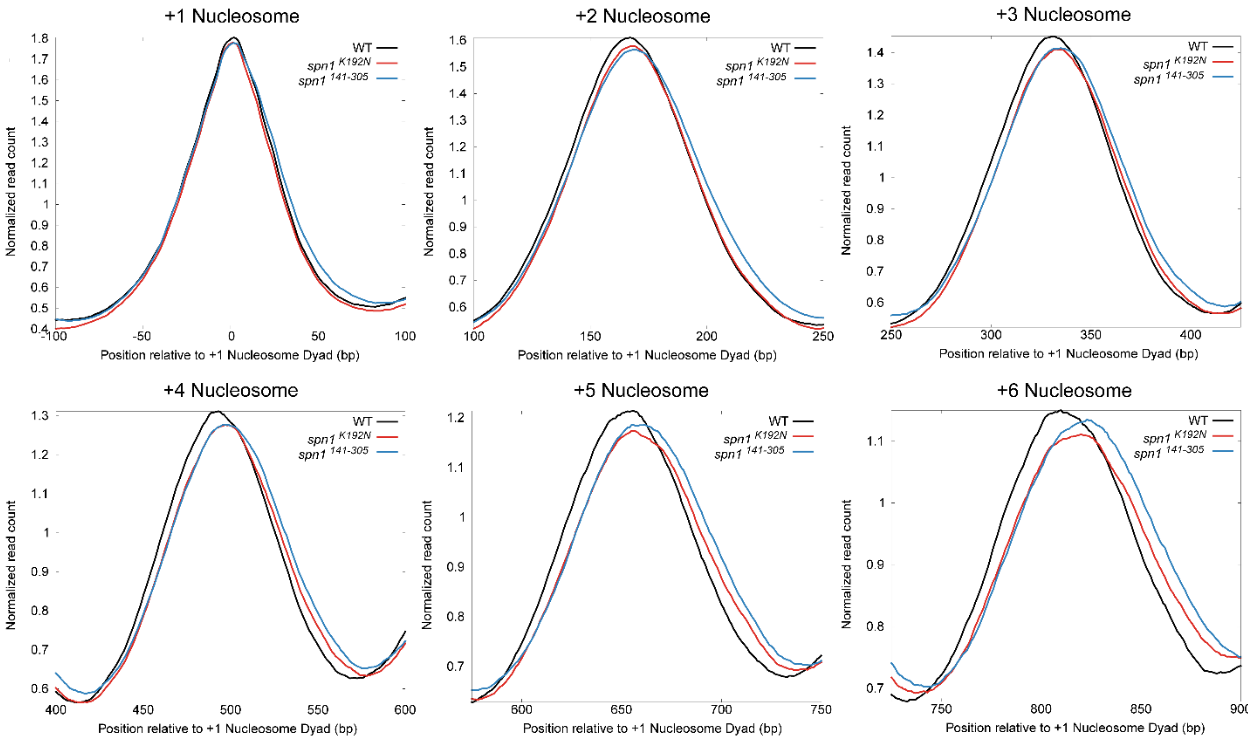

Figure S7: Expression of either ***spn1***^{K192N} **and** ***spn1***$^{141\text{-}305}$ **mutant alleles produced a progressive 3′ downstream shift in nucleosome positioning.** Profiles of MNase protected fragments (80-170 bp) mapped over 4688 genes aligned to the +1 nucleosome dyad in wild-type, *spn1*^{K192N}, and *spn1*$^{141\text{-}305}$ expressing yeast. Panels are rescaled by normalized read count and separated into panels to feature +1 to +6 nucleosome positions.

Figure S8

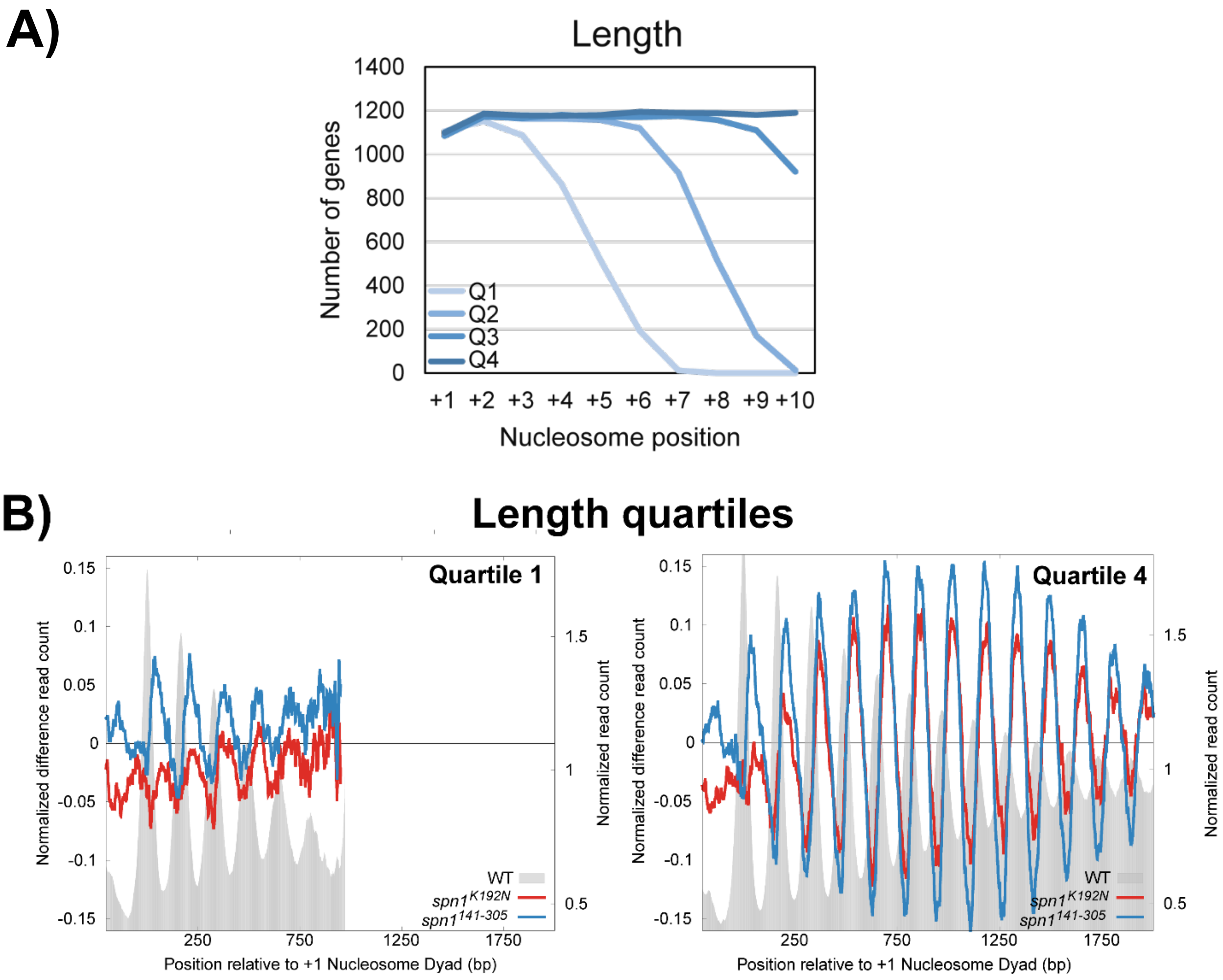

Figure S8: Yeast genes were ranked by gene length to generate quartile gene lists. (A) Count of genes containing mapped nucleosomes at each position across quartiles ranked by length. (B) Difference profile plots between wild-type and mutant samples over 4688 genes binned into quartiles by gene length, with Q1 representing the shortest genes (left panel) and Q4 representing the longest (right panel).

Figure S9

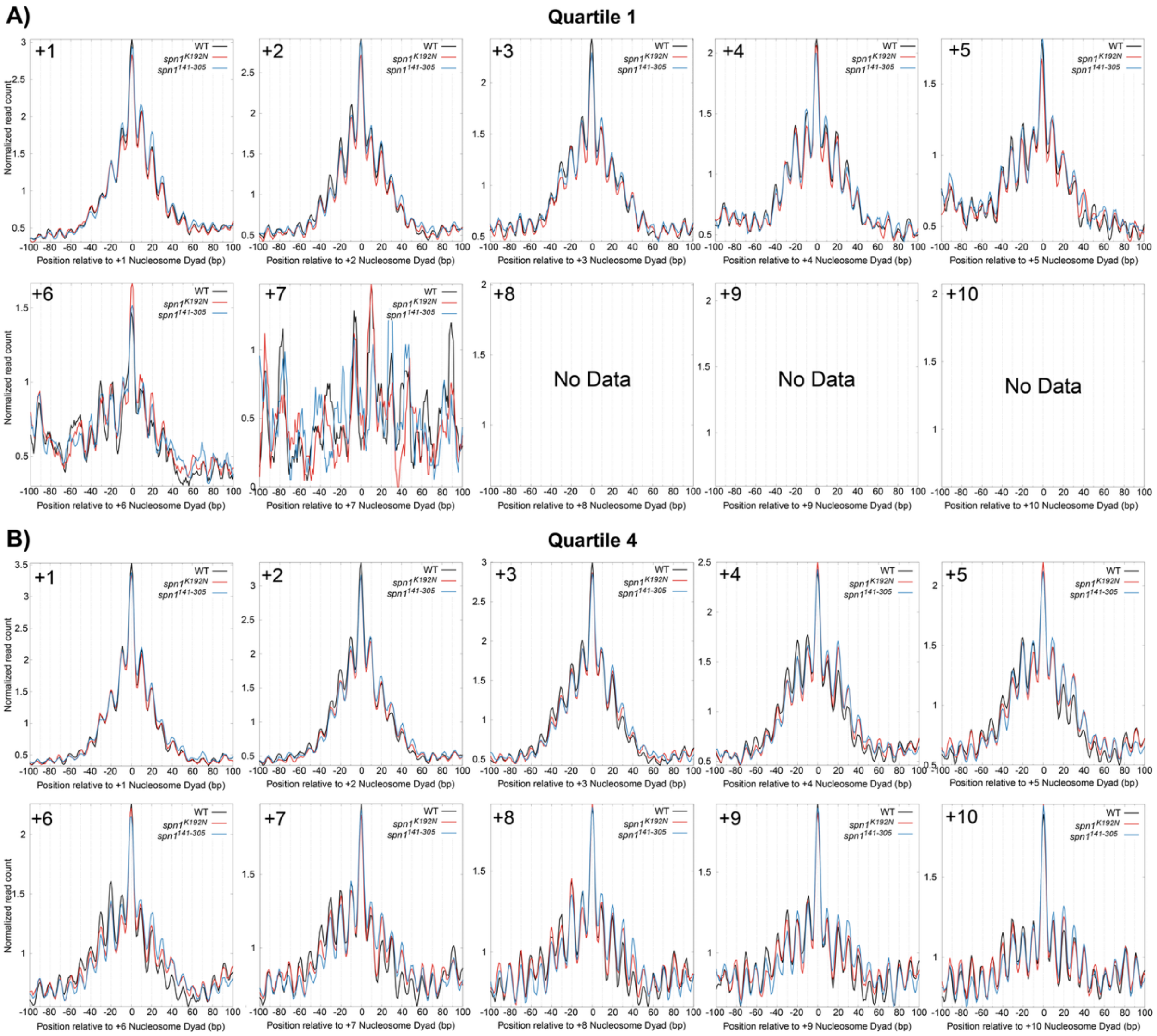

Figure S9: Nucleosomal protections in Spn1 mutants occupy the same rotational positions as wild-type cells, but with higher occupancy at rotational positions downstream of the dyad with a loss of occupancy at positions upstream of the dyad location. Metagene plots of fragment midpoints 142 - 152bp in length mapped to each nucleosome dyad at the (A) shortest and (B) longest gene quartiles ranked by length.

Figure S10

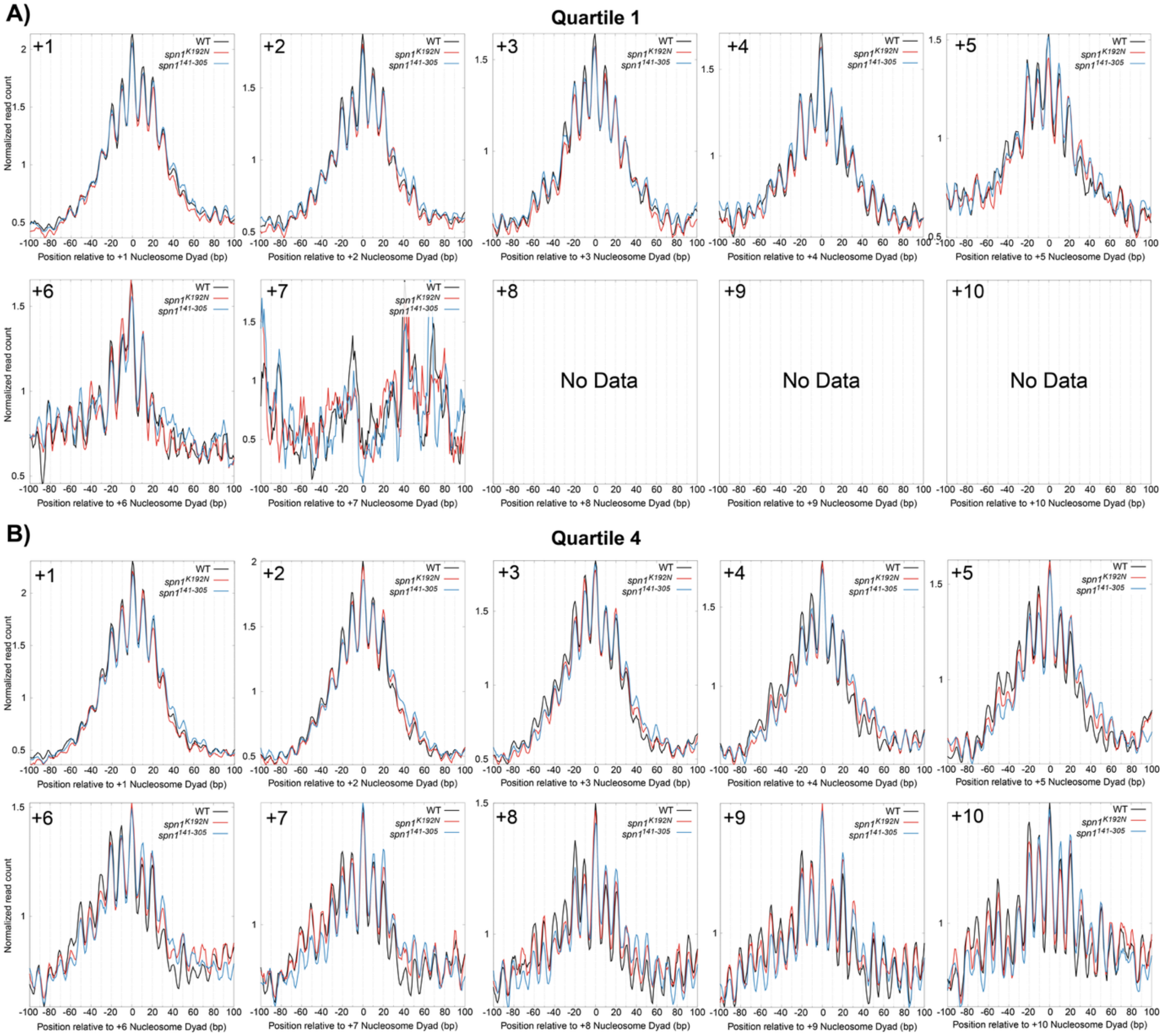

Figure S10: Subnucleosomal protections 102-112 bp in length in Spn1 mutants occupy the same rotational positions as wild-type cells, but with higher occupancy at rotational positions downstream of the dyad with a loss of occupancy at positions upstream of the dyad location. Metagene plots of fragment midpoints 102 - 112bp in length mapped to each nucleosome dyad at the (A) shortest and (B) longest gene quartiles ranked by length.

Figure S11

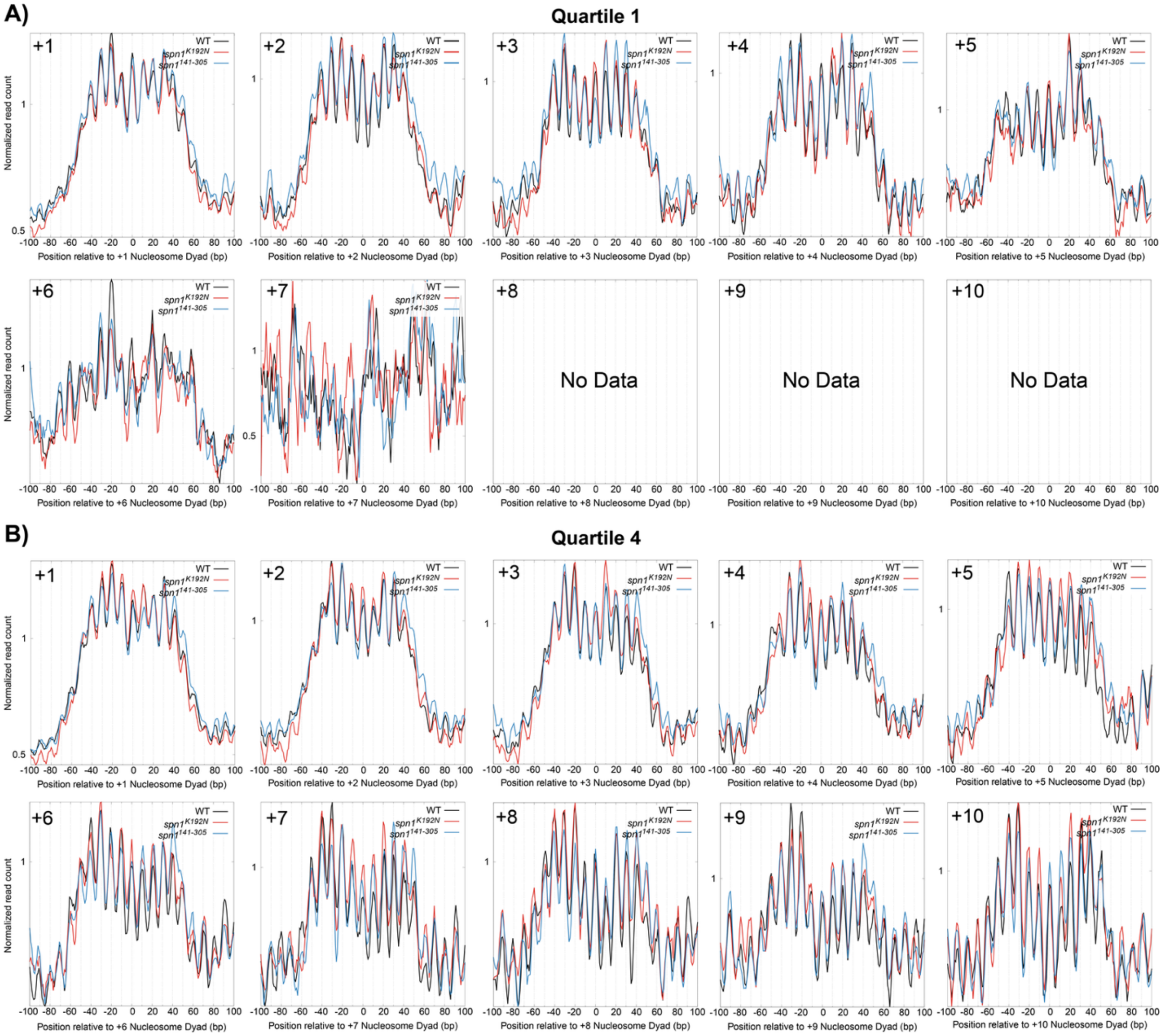

Figure S11: Subnucleosomal protections 61-71 bp in length in Spn1 mutants occupy the same rotational positions as wild-type cells, but with higher occupancy at rotational positions downstream of the dyad with a loss of occupancy at positions upstream of the dyad location in *spn1*$^{141\text{-}305}$. Metagene plots of fragment midpoints 61 - 71bp in length mapped to each nucleosome dyad at the (A) shortest and (B) longest gene quartiles ranked by length.

Figure S12

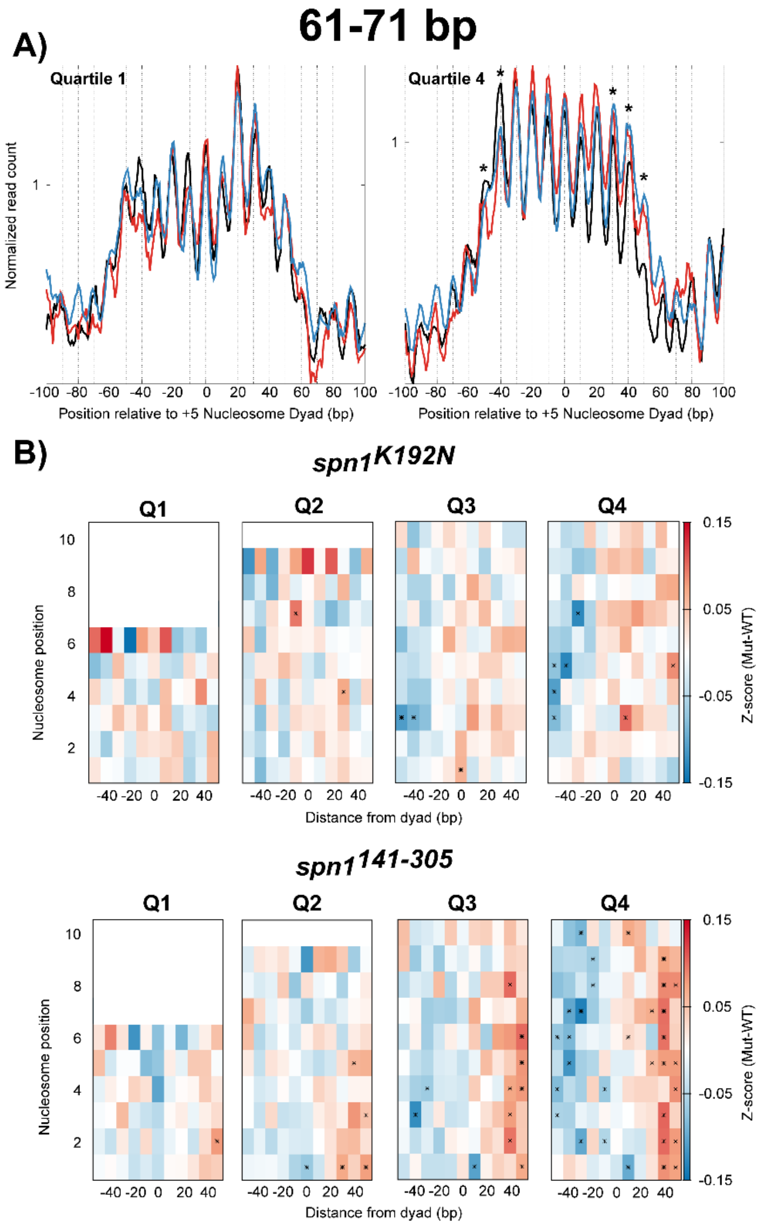

Figure S12: Protected fragments 61-71 bp in length experience little shifts in Spn1 mutant expressing strains. (A) Metagene plots of fragment midpoints 61-71 bp in length mapped to the +5 nucleosome dyad at genes ranked by length. Asterisks indicate rotational positions with statistically significant change in Z-score. (B) Z-score changes in 61-71 bp fragment occupancy at rotational positions surrounding each nucleosome dyad position at genes ranked by length in mutant Spn1 expressing yeast strains compared to WT plotted as a heatmap. Asterisks indicate rotational positions with statistically significant change in Z-score.

Figure S13

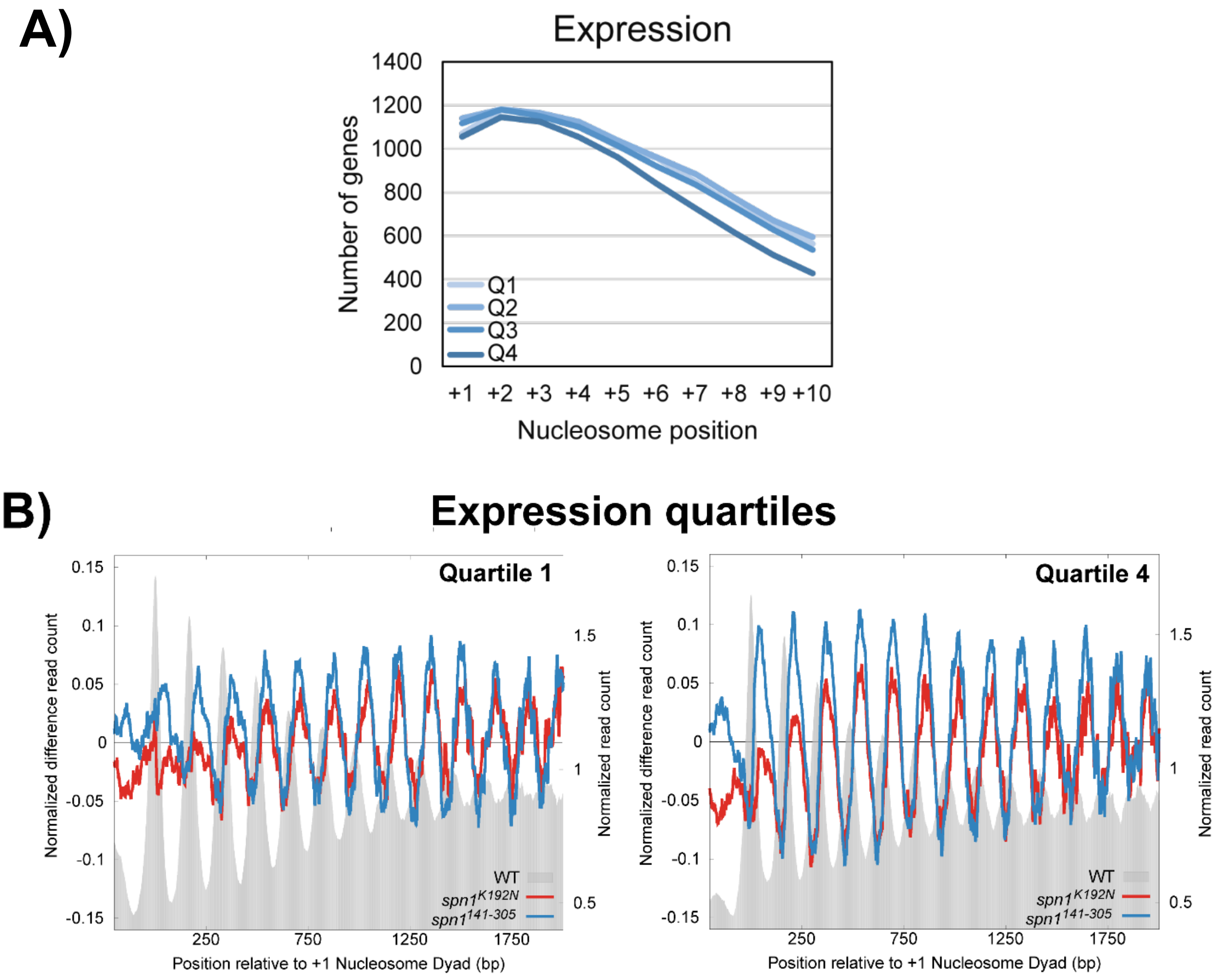

Figure S13: Yeast genes were ranked by published NET-seq data to generate quartile gene lists. (A) Count of genes containing mapped nucleosomes at each position across quartiles ranked by expression. (B) Difference profile plots between wild-type and mutant samples over 4688 genes binned into quartiles by expression, with Q1 representing the least expressed (left panel), and Q4 representing the most expressed genes (right panel).

Figure S14

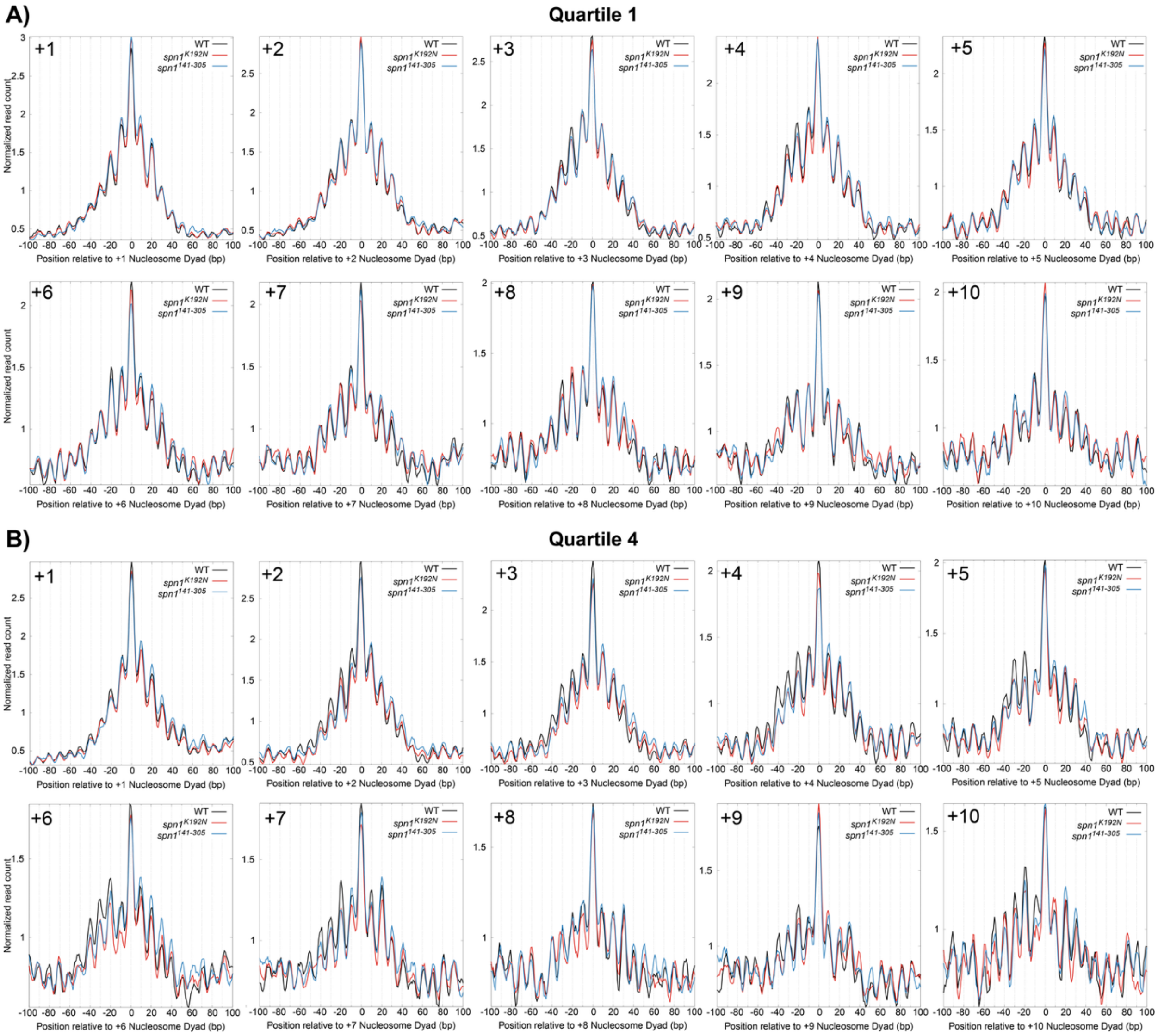

Figure S14: Nucleosomal protections in Spn1 mutants occupy the same rotational positions as wild-type cells, but with higher occupancy at rotational positions downstream of the dyad with a loss of occupancy at positions upstream of the dyad location. Metagene plots of fragment midpoints 142 - 152bp in length mapped to each nucleosome dyad at the (A) lowest and (B) highest gene quartiles ranked by expression.

Figure S15

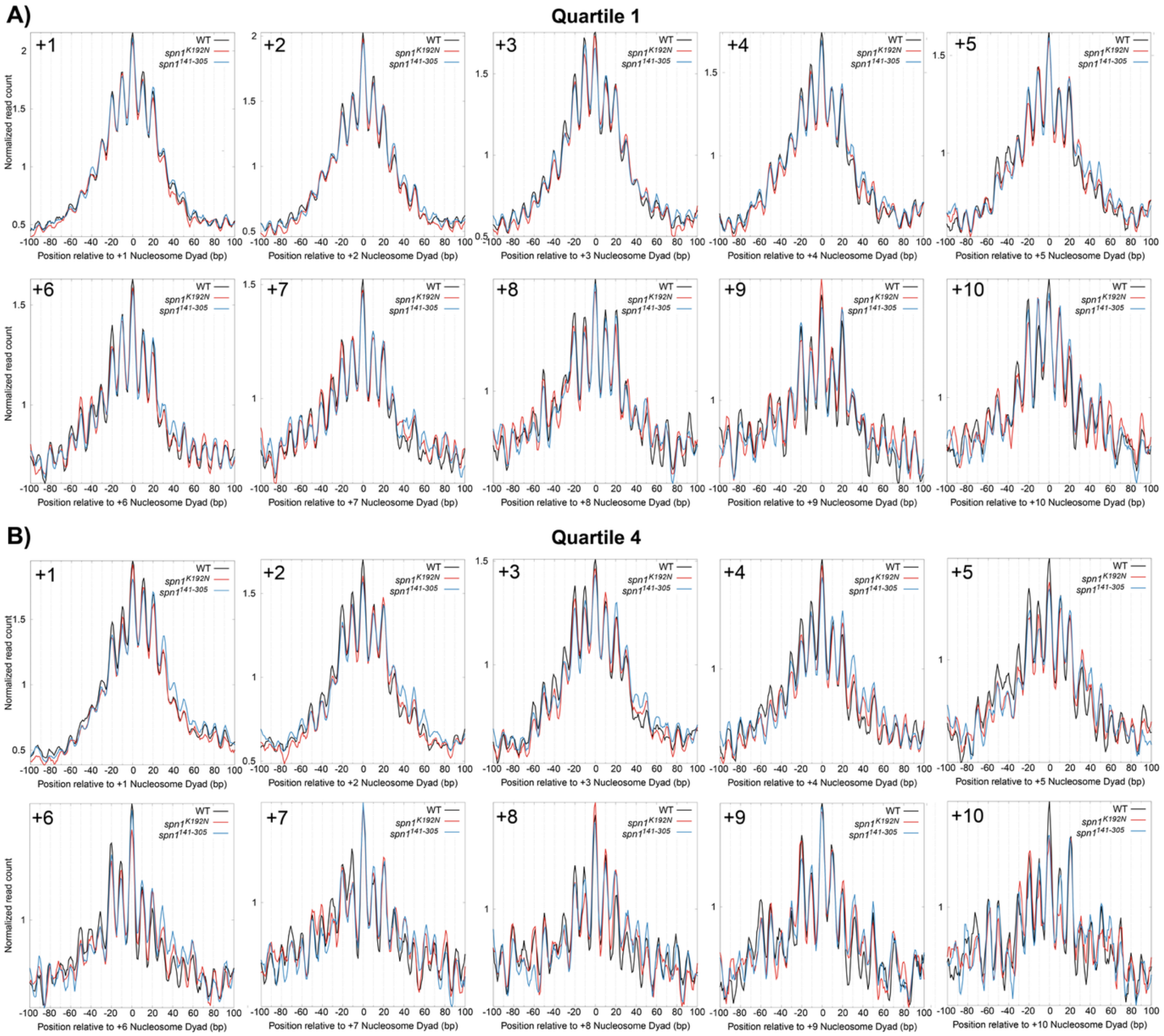

Figure S15: Subnucleosomal protections 102-112 bp in length in Spn1 mutants occupy the same rotational positions as wild-type cells, but with higher occupancy at rotational positions downstream of the dyad with a loss of occupancy at positions upstream of the dyad location. Metagene plots of fragment midpoints 102 - 112bp in length mapped to each nucleosome dyad at the (A) lowest and (B) highest gene quartiles ranked by expression.

Figure S16

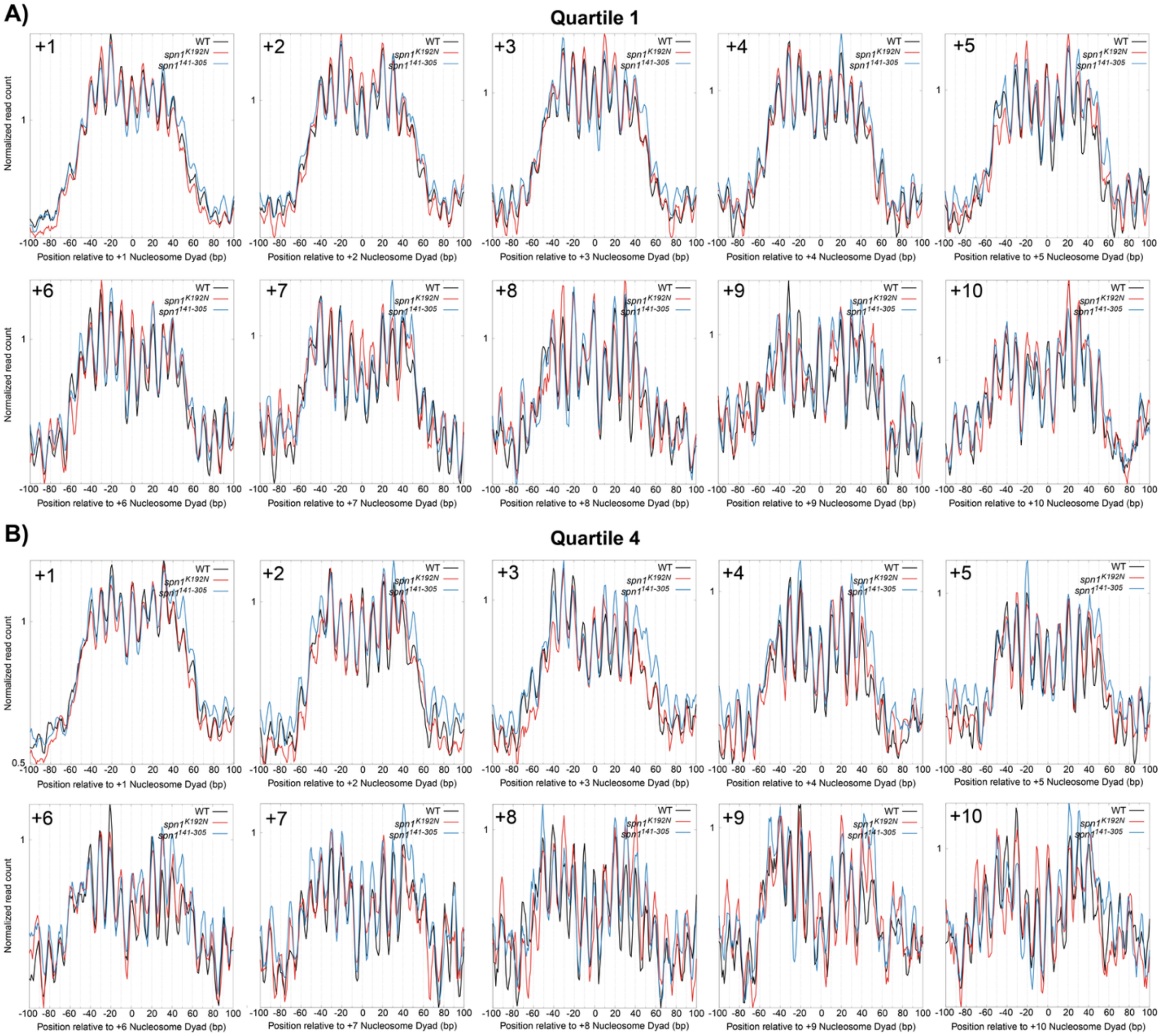

Figure S16: Subnucleosomal protections 61-71 bp in length in Spn1 mutants occupy the same rotational positions as wild-type cells, but with higher occupancy at rotational positions downstream of the dyad with a loss of occupancy at positions upstream of the dyad location in *spn1*$^{141\text{-}305}$. Metagene plots of fragment midpoints 61 - 71bp in length mapped to each nucleosome dyad at the (A) lowest and (B) highest gene quartiles ranked by expression.

Figure S17

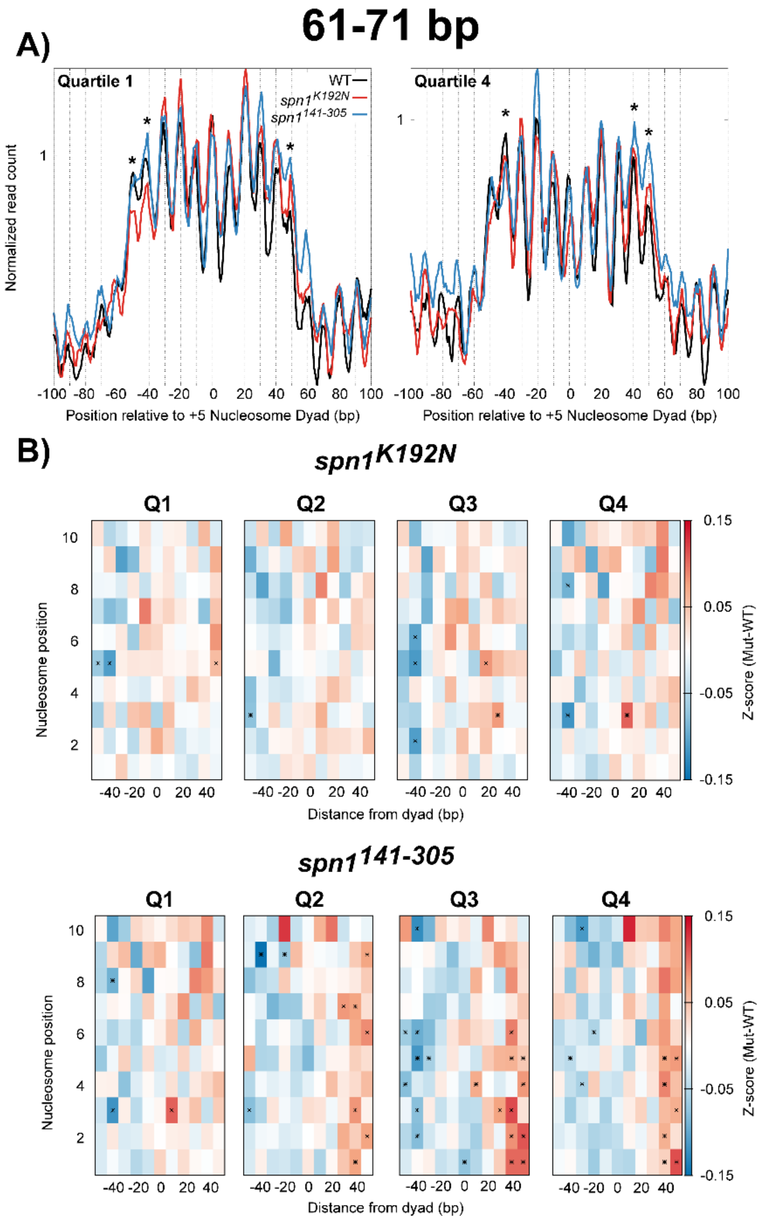

Figure S17. Protected fragments 61-71 bp in length experience little shifts in Spn1 mutant expressing strains. (A) Metagene plots of fragment midpoints 61-71 bp in length mapped to the +5 nucleosome dyad at genes ranked by length. Asterisks indicate rotational positions with statistically significant change in Z-score. (B) Difference heatmaps between wild-type and mutant Spn1 expressing yeast strains scored by changes in 61-71 bp fragment occupancy at rotational positions surrounding each nucleosome dyad position at genes ranked by expression. Asterisks indicate rotational positions with statistically significant change in Z-score.

Figure S18

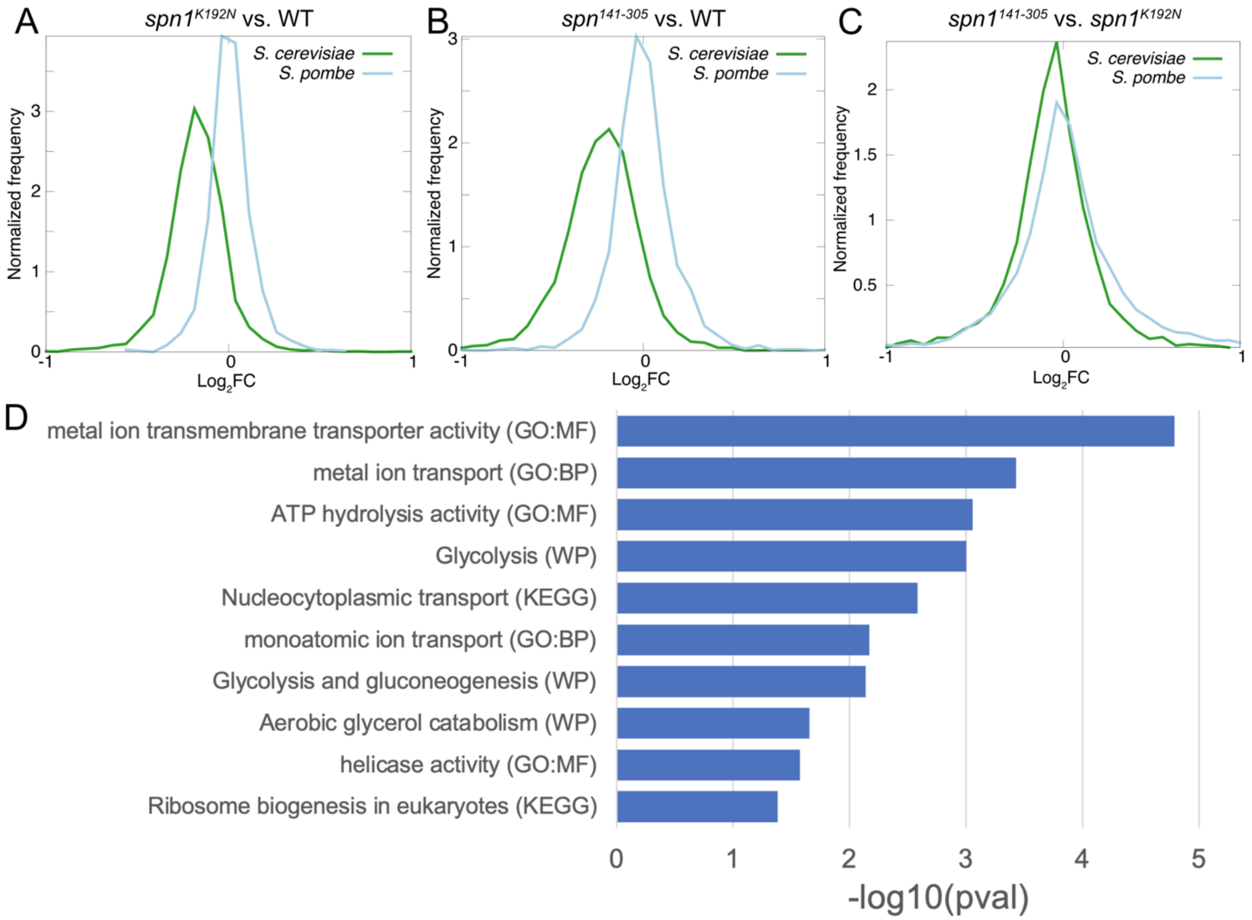

Figure S18. RNA-seq analysis of Spn1 mutants. (**A**) Distribution of $\log_2$(Fold Change) of *S. cerevisiae* and *S. pombe* genes for *spn1*^{K192N} expressing yeast compared to wild-type. (**B**) Same as (**A**) for *spn1*$^{141\text{-}305}$ expressing yeast compared to wild-type. (**C**) Same as (**A**) for *spn1*$^{141\text{-}305}$ expressing yeast compared to *spn1*^{K192N} expressing yeast. (**D**) Top 10 highest enriched terms obtained from gene set enrichment analysis of genes belonging to G1-G4. P-values adjusted for multiple testing correction are plotted on the x-axis. GO:MF denotes Gene Ontology: Molecular Function, GO:BP denotes Gene Ontology: Biological Process, WP denotes Wiki Pathways, KEGG denotes KEGG reactome.

SUPPLEMENTAL REFERENCES

Kubik S, Bruzzone Maria J, Jacquet P, Falcone J-L, Rougemont J, Shore D. 2015. Nucleosome stability distinguishes two different promoter types at all protein-coding genes in yeast. Molecular Cell. 60(3):422-434.

Li S, Almeida AR, Radebaugh CA, Zhang L, Chen X, Huang L, Thurston AK, Kalashnikova AA, Hansen JC, Luger K et al. 2018. The elongation factor spn1 is a multi-functional chromatin binding protein. Nucleic Acids Research. 46(5):2321-2334.

Zhang L, Fletcher AGL, Cheung V, Winston F, Stargell LA. 2008. Spn1 regulates the recruitment of spt6 and the swi/snf complex during transcriptional activation by rna polymerase ii. Molecular and Cellular Biology. 28(4):1393-1403.
